# Signal Integration by Cyclin-Dependent Kinase 8 (CDK8) Module and Other Mediator Subunits in Biotic and Abiotic Stress Responses

**DOI:** 10.3390/ijms22010354

**Published:** 2020-12-31

**Authors:** Leelyn Chong, Xiaoning Shi, Yingfang Zhu

**Affiliations:** State Key Laboratory of Crop Stress Adaptation and Improvement, School of Life Sciences, Henan University, Kaifeng 475001, China; leelyn.chong@yahoo.com (L.C.); shixn555@gmail.com (X.S.)

**Keywords:** CDK8 module, Mediator subunits, biotic stress, abiotic stress, cell signaling

## Abstract

Environmental stresses have driven plants to develop various mechanisms to acclimate in adverse conditions. Extensive studies have demonstrated that a significant reprogramming occurs in the plant transcriptome in response to biotic and abiotic stresses. The highly conserved and large multi-subunit transcriptional co-activator of eukaryotes, known as the Mediator, has been reported to play a substantial role in the regulation of important genes that help plants respond to environmental perturbances. CDK8 module is a relatively new component of the Mediator complex that has been shown to contribute to plants’ defense, development, and stress responses. Previous studies reported that CDK8 module predominantly acts as a transcriptional repressor in eukaryotic cells by reversibly associating with core Mediator. However, growing evidence has demonstrated that depending on the type of biotic and abiotic stress, the CDK8 module may perform a contrasting regulatory role. This review will summarize the current knowledge of CDK8 module as well as other previously documented Mediator subunits in plant cell signaling under stress conditions.

## 1. Introduction

In order to perceive and respond effectively to environmental stresses, plants have developed sophisticated signaling transduction pathways that induce gene expression changes via a complex network of transcription factors (TFs). Phytohormones of abscisic acid (ABA), jasmonic acid (JA), ethylene (ET), and salicylic acid (SA) are known to have extensive crosstalk with other hormones of auxin, brassinosteroid (BR), cytokinins, and gibberellic acid in plant stress response [1,2]. More than 1500 TFs in *Arabidopsis* have been reported to orchestrate the transcriptional control of abiotic stress responses [3]. The regulation of transcription in plant involves RNA polymerase II (RNA Pol II), general TFs, transcriptional activators/repressors, as well as co-regulators such as Mediator. Mediator was initially studied in yeast and subsequently identified to be critical for RNA Pol II-regulated transcription in eukaryotes including mammalian cells and plants through biochemical purification and comparative genomics [4,5].

**Figure 1:**
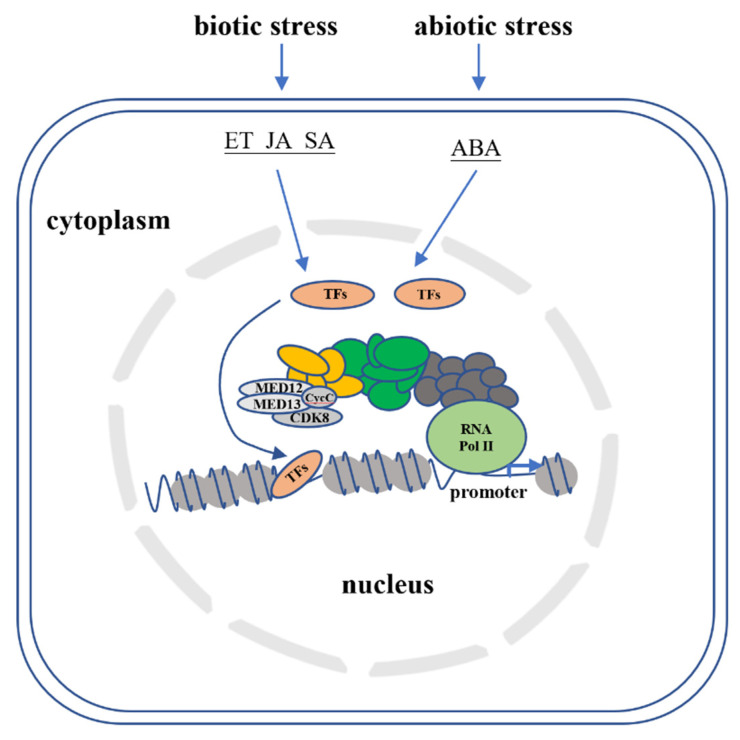
The regulatory functions of Mediator complex in biotic and abiotic stress responses. Biotic and abiotic stresses trigger the plant hormones of ethylene (ET), jasmonic acid (JA), salicylic acid (SA) and abscisic acid (ABA) to activate signaling transduction pathway. A number of transcription factors (TFs) transmit these messages to the transcriptional machinery in the nucleus. Mediator complex functions as a bridge between TFs and RNA polymerase II to precisely regulate the transcription of stress responsive genes.

The Mediator is an evolutionarily conserved large protein complex with multiple components called subunits that transfers upstream regulatory information from activators and repressors to the basal transcriptional machinery in the downstream pathway. A number of Mediator subunits have been identified to play critical roles in plant defense, adaptation, growth and development [6]. More subunits (25–35) are constituted in the Mediator of plant and mammalian cells than in yeast [7]. Mediator subunits of plants have been revealed to involve in various stress-response pathways through genetic analyses. There is also a fourth regulatory kinase module consisting of two Mediator subunits known as MED12 and MED13 as well as a separable kinase unit made up of CDK8 (cyclin-dependent kinase 8) and a C-type cyclin (CycC). CDK8, as its full name implies is a cyclin-dependent kinase and it along with its associated cyclin, CycC, also referred to as the kinase or CDK8-cyclin C module. The whole kinase module is an integral part of the Mediator complex. Studies have indicated that the protein components of the CDK8 kinase module may act as a both positive and negative transcription regulator in cells since CDK8 module was reported to act as a repressor to Mediator when bound to the complex [8,9]. However, there were situations when the CDK8 module worked as an activator on certain genes [10,11]. In plants, the kinase module was initially viewed as a predominant transcriptional repressor in eukaryotic cells by reversibly associating with the core Mediator. Later, evidence that supports the positive regulatory roles of CDK8 module in plants’ transcription has also appeared. In fact, the subunits of MED12 and MED13 were reported recently to serve as conditional positive gene regulators in *Arabidopsis* [12,13,14]. Likewise, the CDK8 subunit is capable of recruiting different TFs to RNA Pol II to regulate multiple signaling pathways in yeast and eukaryotic cells including plant cells [15]. The general working model for the signaling role of CDK8 and other Mediator subunits in plants during biotic and abiotic stresses are illustrated in Figure 1.

*Arabidopsis CDK8*, also named *HUA ENHANCER3* (*HEN3*), was first reported for its regulation of floral organ identity. Thus, it was suggested to contribute to cell differentiation in a multicellular organism [16]. *CDK8* was later found to interact with *Arabidopsis* LEUNIG, a transcription co-repressor [17]. Another name for *Arabidopsis CDK8* is *CDKE1* (cyclin-dependent kinase E1). Studies on the *Arabidopsis regulator of alternative oxidase 1* (*rao1*) mutant which carries a mutation in *CDKE1* further documented that CDKE1 regulates mitochondrial retrograde signaling under H_2_O_2_ and cold stress [18]. Moreover, *CDK8* has been indicated to contribute in drought stress regulation recently. In terms of plant immune responses, *Arabidopsis CDK8* was reported to perform a contrasting regulatory role [19]. Each subunit of the Mediator complex appears to specifically respond to various environmental conditions to different degrees. The significance of the reported subunits, particularly in the CDK8 module, are being explored next for their signaling involvement in biotic and abiotic stresses [20].

## 2. The Role of Mediator Subunits in Biotic Stress Regulation

When plants encounter biotic stress such as pathogen infection, they must either activate and/or suppress many genes. It has been documented that more than 620 genes were induced by common necrotrophic fungi such as *Botrytis cinerea* or *Alternaria brassicicola* [21,22]. Undeniably within minutes after infection, significant transcriptional reprogramming occurs [23,24]. In fact, an extensive number of genes such as lignin synthesis for cell wall fortification, defense proteins that attenuate infection, as well as antimicrobial secondary metabolite production, were induced in plants when a pathogen is sensed. TFs including *WRKY33*, *MYC2*, *ethylene response factor1* (*ERF1*), *ethylene-insensitive3* (*EIN3*) along with a few activators are important for defense gene induction as well as resistance to pathogens [25,26].

Since Bäckström et al. isolated the plant Mediator from *Arabidopsis* back in 2007 [27], Mediator has been established as an essential component of plant defense and development. Its subunit of MED21 was determined by Dhawan et al. to have a role in necrotrophic pathogen defense [28]. *MED25*, also called *PHYTOCHROME AND FLOWERING TIME1*, along with *MED8* were further uncovered by Kidd et al. for their involvement in plant defense [29]. Moreover, *MED25* was also identified to regulate JA and SA-induced gene expression. In 2011, Ou et al. identified eight other TFs that have a direct interaction with *Arabidopsis MED25*. Three of the identified eight genes directly recognize the GCC-box of the promoter of *Plant Defensin 1.2* (*PDF1.2*) gene, a gene that involves in plant defense [30]. Other subunits including *MED33a* and *MED33b*, which correspond to the known *Arabidopsis* mutants *REDUCED EPIDERMAL FLUORESCENCE4* (*REF4*) and *REF4-RESEMBLING1* (*RFR1*), have been reported to regulate the phenylpropanoid pathway [31]. The phenylpropanoid pathway generates products of lignin and anthocyanin, which are essential for plant defense as lignin fortification of cell walls curb pathogen growth [32,33] and anthocyanins are plant pigments that possess antimicrobial activity along with free radical scavenging properties. In fact, during pathogen infection, anthocyanins synthesis incorporates compounds of jasmonates, ABA and sugars to support plant defense [34,35]. The accumulation of anthocyanins is affected by *MED25* [29]. As can be seen from these studies, the involvement of Mediator subunits in biotic stress is a sophisticated network of highly interconnected systems. Mediator subunits interact with defense genes to regulate various signaling transduction pathways that involve phytohormones as well as other hormones that enable the strengthening of plants to fight off pathogen. At the time when these subunits were reported for their roles in biotic stress, the involvement of CDK8 module in environmental disturbances was still unspecified, until recently when more evidence began to emerge to show its importance [36].

## 3. Subunits of the CDK8 Module Play a Role in Regulating Biotic Stress

**Figure 2:**
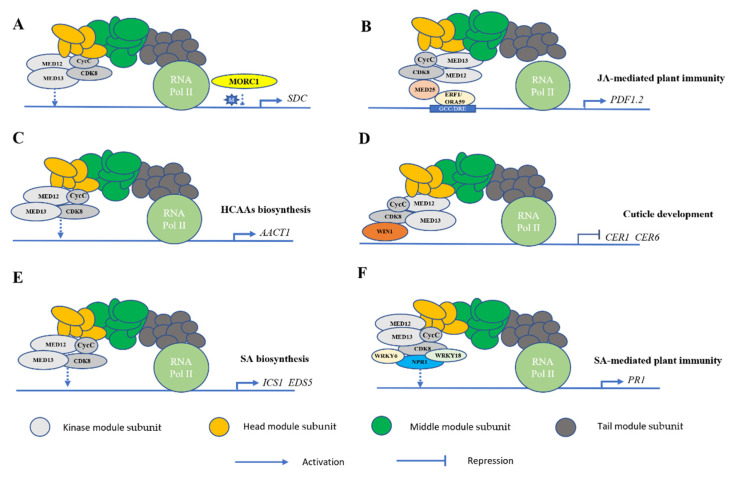
The regulatory functions of CDK8 module in biotic stress response. (**A**) *MED12* and *MED13* reactivate the expression of a silenced DNA methylated *SDC* gene regulated by *MORC1*. (**B**) CDK8, MED12 and MED13 subunits interact with ERF1/ORA59 to regulate the expression of *PDF1.2*. (**C**) *CDK8* is involved in the induced expression of *AACT1* to regulate the biosynthesis of HCAAs for biotic stress response. (**D**) *CDK8* represses the (*ECERIFERUM*) *CER1* and *CER6* gene expression to downregulate cuticle biosynthesis through interacting with WIN1, which binds to the GCC/DRE-box [45]. (**E**) *CDK8* subunit integrates SA signaling through activating the expression of *ICS1* and *EDS5*. (**F**) CDK8, WRKY6, WRKY18, and NPR1 form a complex to regulate the expression of pathogenesis-related (*PR*) genes. Light gray indicates the CDK8 kinase module, yellow indicates the head module, green indicates the middle module, and dark gray indicates the tail module.

Along with the subunits of MED8, MED15, MED16, MED21, and MED25, recent evidence about the substantial role of CDK8 module subunits in the resistance against necrotrophic pathogens has appeared [28,37,38,39]. In response to pathogen infection, plants produce different hormones such as ET, JA and SA [40]. These hormones further activate the expression of defense-related genes. As mentioned previously, very few studies focused on the transcriptional regulation of the CDK8 kinase module in biotic stress. In 2014, Zhu et al. studied the roles of CDK8 subunit in immune responses of *Arabidopsis* [19]. Beside *CDK8,* they further noted that the two other kinase module mutants, *med12* and *med13*, presented signs of disease responses and increased cuticle permeability that were similarly observed in *cdk8* mutant. They proposed that a shared function and structural conservation exist among the kinase module subunits. Since their study was targeting the functional role of CDK8 subunit in biotic stress, they did not mine into the mechanistic roles of *med12* and *med13*. Very recently, *MED12* and *MED13* have been reported for their roles in gene regulation. *MED12* and *MED13* were indicated to participate in the beginning steps of gene transcription and were identified as positive gene regulators under certain conditions. Liu et al. performed mutations of *MED12* and *MED13* and discovered that they suppress *morc1*-reactivated *pSDC:GFP* (*SUPPRESSOR OF DRM1 DRM2 CMT3*) [13] (Figure 2A). *Microrchidia* (*MORC*) is a *GHKL* (gyrase, Hsp90, histidine kinase, MutL)-type ATPase-containing protein that exists in both animal and plant species [41]. It was reported that the silenced DNA methylated genes are reactivated by the mutations of *MORC* proteins in *Arabidopsis* [42]. Apparently, *MED12* and *MED13* are necessary for the expression of genes depleted in active chromatin marks, a chromatin signature shared with *morc1* reactivated loci [43].

As for the subunit of *CDK8*, Zhu et al. have elucidated the functions as well as the underlying molecular and biochemical mechanisms of the Mediator subunit *CDK8* in plant defense. An extensive amount of work has been performed to unfold a contrasting defense function of *CDK8* to two necrotrophic fungi of *A. brassicicola* and *B. cinerea*. Both fungi share similar mechanisms of pathogenesis, virulence, and modes of nutrition. In their investigation of *Arabidopsis*’ biotic response to *A. brassicicola*, they pointed out that *CDK8* regulates the expression of defensin genes including *PDF1.2* and several Ethylene Response Transcription Factors (ERFs) which are reported for disease resistance [19]. This finding was consistent with the observation that *ERF1-* and *OCTADECANOID RESPONSIVE ARABIDOPSIS AP2/ERF59 (ORA59)*-dependent activation of *PDF1.2* expression requires *CDK8* [44] (Figure 2B). Since an interaction was found between CDK8 and MED25, it was suggested that *CDK8* regulates plant immunity through a JA-dependent pathway. Simultaneously, *CDK8* contributes to *Arabidopsis*’ resistance to *A. brassicicola* through direct regulation of *AGMATINE COUMAROYLTRANSFERASE* (*AACT1*) transcription. *AACT1* is involved in the biosynthesis of a class of secondary metabolites called hydroxycinnamic acid amides (HCAAs) (Figure 2C), which are known for their functions in fungal resistance [19]. Therefore, *Arabidopsis* would fail to induce critical defense responses without *CDK8*.

Despite their discovery of the positive regulatory role of *CDK8* in plant immunity, they also uncovered another interesting finding that shows *cdk8* mutant exhibits enhanced resistance to *B. cinerea*. Changes in the cuticle structure and permeability of the *cdk8* mutant were noticed [19]. Therefore, they suggested that the increased cuticle permeability and altered cuticle structure are factors that influenced the *cdk8* mutant’s biotic resistance as cuticles are related to plants’ improved resistance to *B. cinerea* [46,47]. To test this hypothesis, they investigated if *CDK8* could interact with *WAX INDUCER1 (WIN1)* which is an *ERF* family protein known for cuticular wax biosynthesis regulation. In fact, they found that an interaction occurs between CDK8 and WIN1, which indicates that *CDK8* is also involved in cuticle development (Figure 2D). Additionally, they observed that the expression of *CDK8* that was defective in phosphorylation activity failed to rescue the susceptibility of the *cdk8* mutant to *A. brassicicola* while the mutant’s resistance to *B. cinerea* was restored to the wild type (WT); indicating two differential functions of *CDK8* in response to different strains of fungi. They also noted a strong interaction of *CDK8* with two CycCs, thereby supporting an evolutionarily conserved structure of the kinase module in plants [19]. Their overall results from their study have revealed that the Mediator subunit CDK8 as well as the CDK8 module possess multiple regulatory roles in plant defense and development.

It has been revealed that *CDK8* mutations diminished the plant’s resistance against the necrotrophic fungus *B. cinerea*. CDK8 was also demonstrated to interact with MED25 to play a role in the JA signaling [19]. In 2019, another piece of evidence about the subunit CDK8’s role in biotic stress has emerged. Huang et al. discovered *CDK8* through a suppressor screen that used the triple mutant of *camta1/2/3* (*calmodulin-binding transcriptional activator*). Similar to the Mediator complex, CAMTAs are evolutionarily conserved in multicellular eukaryotes in which their roles in transcriptional activity of plants are controversial [48]. They discovered that the mutation of *cdk8* partially suppresses autoimmunity mediated by *camta1/2/3* which includes enhanced resistance against *Hpa Noco2* and SA accumulation levels. Hence, they suggested that *CDK8* positively regulates SA accumulation and systemic acquired resistance (SAR) in *Arabidopsis*. SA is a phytohormone that participates in diverse immune responses including SAR, local defense, and effector-triggered immunity (ETI) whereas SAR is a form of plants’ systemic immune response that becomes activated in uninfected distal parts of plants when a local pathogen infection is detected [49]. SA accumulation is reported to be triggered in both infected and distal tissues after an infection occurs. The study further indicated that CDK8 subunit positively regulates these roles through increasing the expression of SA biosynthesis genes of *ICS1* (*Isochorismate Synthase 1*) and *EDS5* (*Enhanced Disease Susceptibility 5*) as the expression of these two genes was compromised in *cdk8* mutants, preventing the plants to perform immune defense (Figure 2E) [50,51].

Chen et al. later showed that *NONEXPRESSER OF PATHOGENESIS-RELATED GENES (NPR1)* interacts with *CDK8* as well as with WRKY DNA-BINDING PROTEINs such as *WRKY18* to induce the expression of *PATHOGENESIS-RELATED (PR)* genes to promote defense responses in *Arabidopsis* [52]. Additionally, SA was indicated to substantially promote the interactions of these proteins to trigger immune response. *NPR1* has been recognized as a master regulator of SA-mediated local and systemic plant immunity due to its control of the expression of over 2000 genes [53,54]. SAR was found to be included in the *cdk8* and CDK8-associated Mediator mutants. The reduced expression of *NPR1* and *NPR1*-dependent defense genes was observed as well in these mutants compared to WT. *CDK8* positively regulates the expression of *NPR1* through the interaction with both *WRKY6* and *WRKY18* and they are associated with the promoter of *NPR1*. Furthermore, CDK8 interacts with TGACG-Binding (TGA) TFs of *TGA5* and *TGA7*; both are associated with the *PR1* promoter to regulate *PR1* gene expression. Another interesting finding about *CDK8* from their study is that CDK8 recruits RNA Pol II to the promoters and coding regions of *NPR1* and *PR1* to promote their gene expression [52]. From their study, CDK8’s contribution to plant immunity such as SAR is further established. In fact, the study explained that *CDK8* could essentially be the component that helps clarify the relationship between NPR1 and its activation of *PR1* to initiate plant immune response (Figure 2F). Overall, they have demonstrated that under SA influence, CDK8 links TGA TFs and NPR1 (through interacting with *WRKYs*) with RNA Pol II to facilitate *PR1* gene expression.

## 4. Signaling Roles of Mediator Subunits in Abiotic Stress

ABA is a phytohormone that contributes significantly to various developmental processes in the plant’s life cycle. The hormone plays a vital role in the plant’s response to various abiotic stresses including drought, salt, and heat stresses. In addition to biotic stress, Mediator complex, has also been found to serve important roles in the ABA signaling transduction. *MED25* was the first Mediator subunit that was documented to act in response to ABA. *MED25* was found to negatively regulate the ABA signaling pathway as *med25* mutants display an increased sensitivity to ABA during seed germination and early seedling growth. Consistent with its negative role in ABA signaling, *med25* mutant was observed to have an increased expression of ABA-responsive genes in response to ABA treatment compared to WT plants. The transcription of *ABA-INSENSITIVE5* (*ABI5*), a key TF regulating the ABA signaling during seed germination, is induced by ABA, and interestingly, the ABA-induced transcription of *ABI5* was suppressed in *med25* mutants compared to WT. ABI5 protein, however, accumulated at higher abundance in *med25* mutants compared to WT, indicating that *MED25* may negatively regulate *ABI5* at post-transcriptional level. Chromatin immunoprecipitation (ChIP) experiments further indicated that *MED25* was highly enriched at the promoters of *ABI5* downstream genes but this enrichment was decreased upon ABA treatment. A direct interaction was noticed between MED25 and ABI5 and this interaction was attenuated by ABA, which was in accordance with the negative impacts of *MED25* on the *ABI5*-regulated ABA responses [38]. MED25 may act as a critical regulator in hormones crosstalk between JA, ethylene and ABA signaling due to its interaction with MYC2 and several TFs in plants. In addition to *MED25*, the head module subunit *MED18* has also been reported to have a role in ABA signaling. Opposite to *med25*, *med18* mutants are more insensitive to ABA at seed germination and early growth stages, similar to *abi4* and *abi5* mutants. Interestingly, the ABA-induced expressions of *ABI4* and *ABI5* are much lower in *med18* mutants than those in WT, suggesting that the transcription of *ABI4* and *ABI5* are positively regulated by *MED18*. ChIP-qPCR further revealed that *MED18* is recruited to the ABI4 binding site on the *ABI5* promoter under both mock and ABA treatments [55]. The physical interaction between MED18 and TF ABI4 further reinforces that *MED18* regulates ABA response and expression of *ABI5* by interacting with ABI4. *MED12* of the CDK8 module was also found recently to contribute to abiotic stress by blocking transient gene upregulation after light treatment as it has interactions with genes that are responsive to environmental stimuli such as light and radiation [13].

## 5. Signaling Roles of CDK8 Subunit in Abiotic Stress

**Figure 3:**
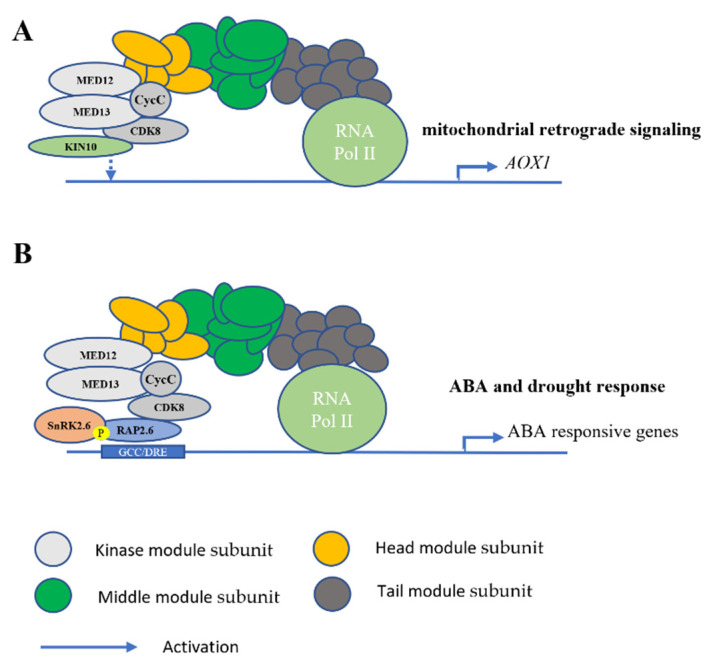
The regulatory functions of CDK8 subunit in abiotic stress response. (**A**) CDK8 cooperates with KIN10 to control the expression of *AOX1* to regulate mitochondrial retrograde signaling. (**B**) CDK8 along with phosphorylated proteins of RAP2.6 and SnRK2.6 bind to the GCC/DRE-box to initiate the transcription of ABA responsive genes in response to ABA and drought stress. Light gray indicates the CDK8 kinase module, yellow indicates the head module, green indicates the middle module and dark gray indicates the tail module.

In 2013, Ng et al. studied cyclin-dependent kinase E1/Regulator of AOX1a 1 (*CDKE1/RAO1*) to understand how the induction of alternative oxidase (*AOX*) is integrated into the general regulatory context of the cell. To their surprise, *CDKE1/RAO1* did not interact directly with any other cyclin components even though *CDKE1* is a cyclin-dependent kinase. *rao1* mutants in the study did not show an alternation in *ABI4* gene expression under normal conditions but its gene expression was changed in a complex *aox1a* knock-out mutant. In fact, they noticed that only 119 transcripts genome-wide were significantly changed by 2.5-fold greater than the *pAOX1a:LUC* under normal conditions in both *rao1–1* and *rao1–2* [18]. Additionally, KIN10 was found to directly interact with CDK8 to coordinately regulate overlapping target genes in response to mitochondrial stress (Figure 3A). They suggested this change was due to the integrated network regulated by direct phosphorylation events associated with the Mediator complex and not any secondary effect caused by stress responsive TF dysregulation.

They also conducted global transcriptional analyses and found that transcripts relating to both growth and stress are affected in the *rao1* mutant background but in opposite ways. It appears that *CDK8* plays a role in initiating cell wide stress responses, protein metabolism and protein synthesis, which are the essential building blocks for cell division and growth. Photosynthetic components, however, are switched off. Their study demonstrated that *CDKE1/RAO1* has a role as it can integrate cellular responses to environmental signals for cell division or elongation. Furthermore, they have shown that *CDKE1/RAO1* essentially serves as a sensitive relay between specific stress-induced TFs that are bound to the promoter and RNA Pol II, therefore it can directly regulate transcription under stress [18]. In general, the study has indicated that plants can utilize *CDKE1/RAO1* as a mechanism to switch between growth and stress responses when responding to different environmental conditions.

Drought and cold stresses are some of the environmental challenges that prevent plant growth and development. In order to overcome stress, plants utilize various strategies including regulating signaling transduction pathways to respond to adverse environmental conditions. *MED16*, *MED25*, *MED14*, and *MED2* are notable for their roles in cold stress regulation. At the time when these subunits were known for their involvement in abiotic stress responses, the function of *CDK8* in abiotic stress was unidentified. It was not until 2020 that Zhu et al. reported that *CDK8* positively modulates drought response in *Arabidopsis*. Since *CDK8* is known for possessing kinase activity, they thought that it would be interesting to explore its potential in the ABA signaling pathway in which phosphorylation is involved. Through utilizing genetic, transcriptomic, and biochemical approaches, it was solidified that CDK8 associates with RAP2.6 and SnRK2.6 to positively regulate the transcription of ABA-responsive genes (Figure 3B). From their study, they have discovered that *CDK8* mutation in *Arabidopsis* results in higher stomata density, impaired stomatal aperture as well as reduced tolerance to drought. Consistently, over-expression of *CDK8* in *Arabidopsis* enhances drought tolerance. They have also observed improved cuticle permeability and thinner cutin in *cdk8* mutants and, thus, they suggested that *CDK8* may possibly regulate drought response through multiple mechanisms [56]. CDK8 was revealed to have a direct interaction with ERF/AP2 type TFs WIN1 (WAX INDUCER1) and RAP2.6 [19,56]. As mentioned previously, *WIN1* is a key regulator of cuticle wax biosynthesis and *RAP2.6* is an abiotic stress responsive gene. It was very likely that *CDK8* upregulates cutin biosynthesis and wax accumulation through interacting with WIN1. Interestingly, *WIN1* may also participate in abiotic stress response as its expression is significantly induced by various abiotic stresses. Their study suggested that WIN1 can bind to the GCC-box and DRE element sequences to activate several stress-responsive genes, suggesting a potential function of CDK8-WIN1 interaction in drought response. Their findings also uncovered another strategy which *Arabidopsis* utilizes CDK8 to cooperate with RAP2.6-SnRK2.6 complex to facilitate the immediate transcription of stress-responsive genes in drought.

## 6. Conclusions and Perspectives

When plants encounter biotic stress, they tackle the challenges using their built-in defense mechanisms and/or triggering defense-related signaling pathways. Activation of these responses often leads to the altered expression of various defense genes. Some of these defense genes are responsible for cuticle formation as well as for the biosynthesis and modification of cell wall [53,57]. Other defense genes are related to pathogenesis. Moreover, genes involved in various hormone signaling pathways have also been reported to change when plants respond to biotic stress [58,59]. As an essential player in the transcriptional regulation of gene expression, it was no surprise to find that the subunits of Mediator have connections with stress mitigation. The current reports of the core Mediator subunits along with the CDK8 module in plant cell signaling has revealed that Mediator has contrary roles in gene regulation when responding to stress.

The CDK8 module, consisting of CDK8, MED12, MED13 and C-type cyclins, is an important part of the Mediator complex. This kinase module is dissociable during transcription and thus initially considered to be a negative regulator of transcription [60]. Increasing evidence, however, indicated that the module can also stimulate transcription [61,62], thereby suggesting that *CDK8* can both positively and negatively regulate transcription as indicated in Table 1. It is believed that the positive regulatory roles of *CDK8* in transcription are either performed through the phosphorylation of TFs which would lead to protein degradation in some reported cases or through the promotion of RNA Pol II elongation [63]. *CDK8* was revealed to regulate plant immunity through kinase dependent and independent functions in Arabidopsis. It is worth investigating if CDK8 may potentially interact with Arabidopsis signal responsive1 (AtSR1)/CAMTA3 to regulate plant growth during plant defense against pathogens as *AtSR1* has been indicated to involve in the regulation of auxin- and BRs-related pathways as well as in the suppression of genes elicited by pathogen attack through binding to the “CGCG” containing CG-box in target gene promoters (51). Aside from serving a role in plant immunity, *Arabidopsis CDK8* module also has been shown to play a role in abiotic stresses. Other subunits such as MED12 and MED13 in the CDK8 module are reportedly served as conditional positive regulators in biotic stress. Subunits in the Mediator complex including *MED25* contributes to drought and salt tolerance in *Arabidopsis* by physically interacting with dehydration-responsive element-binding protein 2a (DREB2a), zinc-finger homeodomain protein 1 (ZFHD1), and a MYB TF through the ACID domain [64].

More evidence is emerging regarding the signaling roles of Mediator subunits in biotic and abiotic stresses. The involvement of these subunits in plant defense indicates the capability of the Mediator to accommodate new pathogen resistances in plants. Not only that, recent reports about these subunits in abiotic stresses also indicate the ability of Mediator to assist plants in acclimating in harsh environment [64,65]. The diverse roles depicted about the Mediator, especially its potential with various TFs interaction in regulating abiotic and biotic stress signaling, suggested a unique capacity of plants to recognize new factors to generate effective responses to adverse conditions. Since the subunit(s) of Mediator can interact with various TFs, it is comprehensible to see it serving as a hub for cross-linking many hormones and other signaling pathways to regulate stress responses. It is very possible that the subunits within the Mediator will cooperate with each other when the plants face new environmental challenges. Moreover, the function of Mediator subunits that respond to biotic and abiotic stresses may also be expanded with the possibility of them acquiring other new subunit(s) to recognize new regulatory proteins in plant survival against unfavorable circumstances.

## Figures and Tables

**Table 1:** Genes regulated by the CDK8 module in *Arabidopsis*.

Subunit	Genes	Regulation	Functions	Reference
CDK8	*ICS1*, *EDS5*	positive	SA biosynthesis	[12]
MED12, MED13	*SDC*	positive	unknown	[13]
CDK8	*PDF1.2*, *ERF*	positive	JA-mediated plant immunity	[19]
CDK8	*NPR1*, *PR1*	positive	SA-mediated plant immunity	[52]
CDK8	*AACT1*	positive	Biosynthesis of defense metabolites HCAAs	[19]
CDK8	*CER1*, *CER6*	negative	Cuticle development	[19]
CDK8	*AOX1*	positive	Mitochondrial retrograde signaling	[18]
CDK8	*COR15A*, *RD29B*, *DREB2A*	positive	ABA and drought responses	[56]
